# Subacute granulomatous thyroiditis: histopathological spectrum, perineural inflammation, and coexisting lesions

**DOI:** 10.1590/1806-9282.20260876

**Published:** 2026-10-12

**Authors:** Muhammed Yunus Akar, Hasan Gucer

**Affiliations:** 1 Recep Tayyip Erdogan University, Faculty of Medicine, Department of Pathology – Rize, Türkiye.

**Keywords:** De Quervain thyroiditis, Thyroiditis, subacute, Peripheral nerves, Inflammation, Thyroid diseases, Thyroid cancer, papillary, Papillary carcinoma

## Abstract

**OBJECTIVE:**

Subacute granulomatous thyroiditis is a self-limited inflammatory disorder and the most common cause of painful thyroid disease. Surgically treated cases offer a unique opportunity to explore its histopathological spectrum and co-occurring thyroid pathologies.

**METHODS:**

We retrospectively analyzed 44 thyroidectomy specimens diagnosed with subacute granulomatous thyroiditis between 2009 and 2019, evaluating clinical data and detailed histopathological features including inflammation extent and associated thyroid lesions.

**RESULTS:**

The cohort comprised 36 females and 8 males (mean age 48.1±8.9 years). Preoperative fine-needle aspiration cytology was performed in 68.2% but correctly suggested subacute granulomatous thyroiditis in only one case. Surgery was most indicated for multinodular goiter (63.6%). All cases exhibited confluent folliculocentric granulomas; additional findings included fibrosis (90.9%), neutrophilic infiltration (63.6%), follicular atrophy (63.6%), and perineural inflammation (63.6%). Coexisting follicular nodular disease was present in 88.6% and chronic lymphocytic thyroiditis in 15.9%. Concomitant malignancy was identified in 13 cases (29.5%), predominantly subcentimeter papillary thyroid carcinomas, with no significant clinicopathological associations (p>0.05).

**CONCLUSION:**

Subacute granulomatous thyroiditis demonstrates broader pathological significance than traditionally recognized. Perineural inflammation may contribute to pain generation, and the elevated malignancy rate most likely reflects incidental detection due to extensive sampling. These findings highlight diagnostic pitfalls and underscore the importance of careful pathological assessment.

## INTRODUCTION

Subacute granulomatous thyroiditis (SGT), also known as de Quervain thyroiditis, is a self-limited post-viral inflammatory disorder of the thyroid gland and the most common cause of painful thyroid disease. It predominantly affects middle-aged women, and most patients are managed conservatively without surgical intervention^1^.

Despite well-recognized clinical features, the histopathological spectrum of SGT remains relatively underexplored, as surgical specimens are uncommon and typically limited to diagnostically challenging cases^2^. SGT can clinically, radiologically, and macroscopically mimic thyroid malignancy^3–5^, and its coexistence with nodular thyroid disease may obscure the detection of underlying neoplasms.

Although the classical hallmark of SGT is granulomatous inflammation with multinucleated giant cells, additional features such as fibrosis, neutrophilic infiltration, and follicular destruction vary according to disease stage. Notably, two aspects remain incompletely understood: the mechanism underlying its characteristic pain and its relationship with coexisting thyroid malignancies. Inflammatory processes involving peripheral nerves have been implicated in pain generation in various organ systems^6^, yet this mechanism has not been systematically evaluated in SGT. Similarly, the increasing detection of incidental subcentimeter papillary thyroid carcinomas raises questions about whether any reported association with SGT reflects a true biological relationship.

This study aimed to provide a comprehensive clinicopathological evaluation of SGT in thyroidectomy specimens, with particular emphasis on (i) detailed histopathological characterization including less commonly described findings; (ii) the presence and potential significance of peripheral nerve inflammation; and (iii) the frequency and nature of coexisting benign and malignant thyroid lesions. We also sought to highlight diagnostic pitfalls of preoperative fine-needle aspiration cytology (FNAC).

To our knowledge, no previous surgical pathology series has systematically evaluated the full histopathological spectrum of SGT, including perineural inflammation and associated thyroid lesions. Furthermore, the mechanism underlying thyroid pain and the significance of coexisting thyroid carcinoma remain incompletely understood. These knowledge gaps formed the rationale for the present study.

## METHODS

### Case selection and study design

A retrospective search of the hospital information management system identified 44 cases diagnosed with SGT among total thyroidectomy specimens received at the Medical Pathology Laboratory of Recep Tayyip Erdoğan University Training and Research Hospital between 2009 and 2019. Ethical approval was obtained from the Institutional Ethics Committee.

All available H&E-stained slides were retrieved and re-evaluated. Although the original cytological reports were issued prior to the 2023 Bethesda System, all cases were retrospectively reclassified according to this updated framework to ensure terminological consistency and facilitate comparability with current literature.

Perineural inflammation was defined as the presence of inflammatory cells circumferentially surrounding or infiltrating the perineurium of identifiable peripheral nerve twigs. Vasculitic-like changes were defined as dense perivascular lymphocytic inflammation involving small- to medium-sized intrathyroidal vessels, with focal mural inflammatory infiltration and endothelial swelling, in the absence of fibrinoid necrosis or thrombosis, thereby distinguishing this reactive pattern from a true necrotizing vasculitis. The fibrosis score was graded semi-quantitatively as follows: 0, no fibrosis; 1 (mild), focal thin fibrous septa involving less than 25% of the examined tissue; 2 (moderate), fibrous bands involving 25–50% of the tissue without distortion of the adjacent follicular architecture; and 3 (severe), broad, confluent fibrosis involving more than 50% of the tissue with architectural distortion. The SGT inflammation score reflected the extent of intraparenchymal granulomatous inflammation and was graded as Score 1 (mild), involving less than 30% of the parenchyma; Score 2 (moderate), involving 30–74%; and Score 3 (severe), involving 75% or more. Neutrophilic infiltration was recorded as present when neutrophils were identified within or at the periphery of granulomatous foci or forming microabscesses, a feature regarded as suggestive of early stage disease. Intragranulomatous necrosis was defined as central eosinophilic, karyorrhectic, or coagulative necrotic debris within granulomatous foci, distinguished from simple colloid degeneration by the presence of necrotic inflammatory cell debris and loss of the follicular epithelial outline. Oncocytic change was defined as the presence of follicular epithelial cells with abundant granular eosinophilic cytoplasm due to mitochondrial accumulation, involving non-granulomatous follicles adjacent to the inflammatory process.

### Statistical analysis

Statistical analyses were performed using SPSS Statistics version 22.0 (IBM Corp., Armonk, NY, USA). Descriptive statistics were presented as numbers and percentages for categorical variables and as mean, standard deviation, median, minimum, and maximum values for continuous variables. Comparisons between two independent groups were performed using Student's t-test when the assumption of normal distribution was met. Categorical variables were compared using the chi-square test or Fisher's exact test, as appropriate. A two-sided p-value of less than 0.05 was considered statistically significant.

## RESULTS

### Clinical and laboratory findings

The cohort consisted of 36 females (81.8%) and 8 males (18.2%), with a mean age of 48.1±8.9 years (range: 32–71). Among patients with available thyroid function data, 80% were euthyroid, 14.2% had subclinical hyperthyroidism, and 5.8% had overt hyperthyroidism.

FNAC was performed in 30 patients (68.2%). Cytological categories included nondiagnostic (6 cases, 20.0%), benign (9 cases, 30.0%), atypia of undetermined significance (3 cases, 10.0%), follicular neoplasm (5 cases, 16.7%), and suspicious for malignancy (7 cases, 23.3%). Only one case demonstrated cytological findings consistent with SGT. Surgical indications were multinodular goiter (28 cases, 63.6%), suspicious cytology (7 cases, 15.9%), follicular neoplasm (5 cases, 11.4%), coexisting parathyroid adenoma (3 cases, 6.8%), and refractory severe pain (1 case, 2.3%).

### Macroscopic and histopathological findings

Bilateral pallor was observed in 14 cases (31.8%) and unilateral pallor in 11 cases (25.0%), whereas 19 cases (43.2%) showed no distinct macroscopic abnormality beyond nodular disease. The mean thyroid weight was 28.6±14.2 g (range: 6–76 g).

Microscopically, inflammation was classified as diffuse (17 cases, 38.6%), nodular (13 cases, 29.5%), or mixed (14 cases, 31.9%). SGT involvement was confined to parenchyma in 39 cases (88.6%), while 5 cases (11.4%) showed extension into areas of follicular nodular disease.

Granulomas were identified in all cases (100%), typically follicle-centered and coalescent. Multinucleated giant cells were present in all cases.

Neutrophilic infiltration, suggestive of early stage disease, was observed in 28 cases (63.6%).

Intragranulomatous necrosis was rare (2 cases, 4.5%) and limited to focal, submillimetric coagulative necrosis.

The extent of intraparenchymal inflammation was <30% in 17 cases (38.6%), 30–74% in 13 cases (29.5%), and ≥75% in 14 cases (31.9%). A semiquantitative scoring system was applied as follows: Score 1 (Mild, <30%), Score 2 (Moderate, 30–74%), and Score 3 (Severe, ≥75%), as defined for the purposes of this study.

Oncocytic changes were present in five cases (11.4%), frequently in association with chronic lymphocytic thyroiditis.

Perineural inflammatory infiltration was observed in 28 cases (63.6%), while vasculitic-like changes were present in 16 cases (36.4%) (Figure 1).

**Figure 1:**
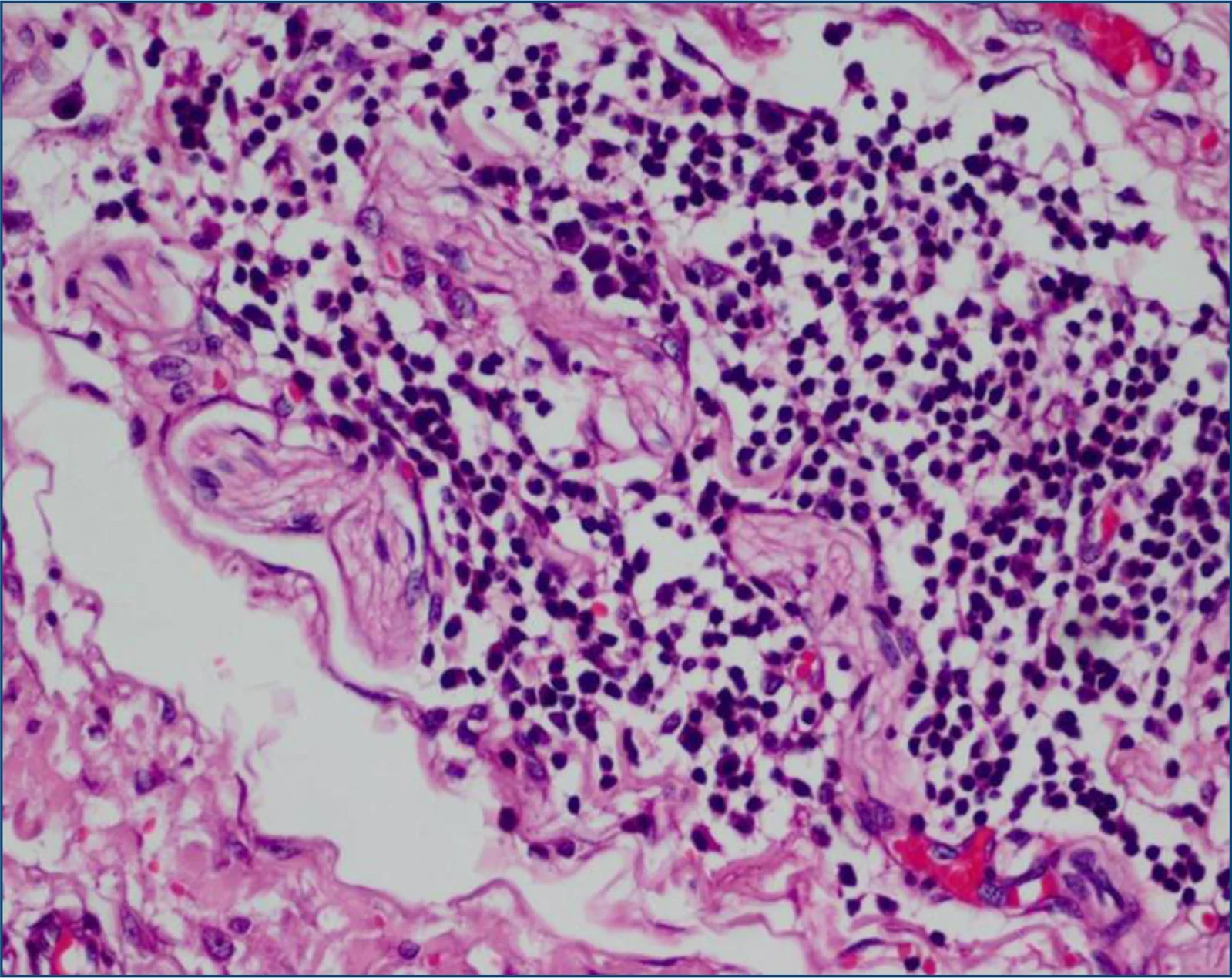
Lymphoplasmacytic inflammatory cell infiltration within and around peripheral nerves (hematoxylin & eosin, ×400).

Fibrosis was a prominent feature, with severe fibrosis observed in 25 cases (56.8%), indicating advanced-stage disease.

Atrophic follicles within inflamed fibromyxoid stroma were identified in 28 cases (63.6%).

Inflammation in perithyroidal soft tissue was observed in 22 cases (50.0%) and edema in 20 cases (45.5%). These findings did not consistently overlap.

Follicular nodular disease (FND) was present in 39 cases (88.6%). Follicular adenoma was identified in six cases (13.6%) and chronic lymphocytic thyroiditis in seven cases (15.9%).

Concomitant malignancy was identified in 13 cases (29.5%), including classic subtype subcentimeter papillary thyroid carcinoma (7 cases, 15.9%), minimally invasive encapsulated follicular variant papillary carcinoma (3 cases, 6.8%), tall cell subtype subcentimeter papillary thyroid carcinoma (2 cases, 4.5%), and infiltrative follicular subtype papillary carcinoma (1 case, 2.3%) (Figure 2).

**Figure 2:**
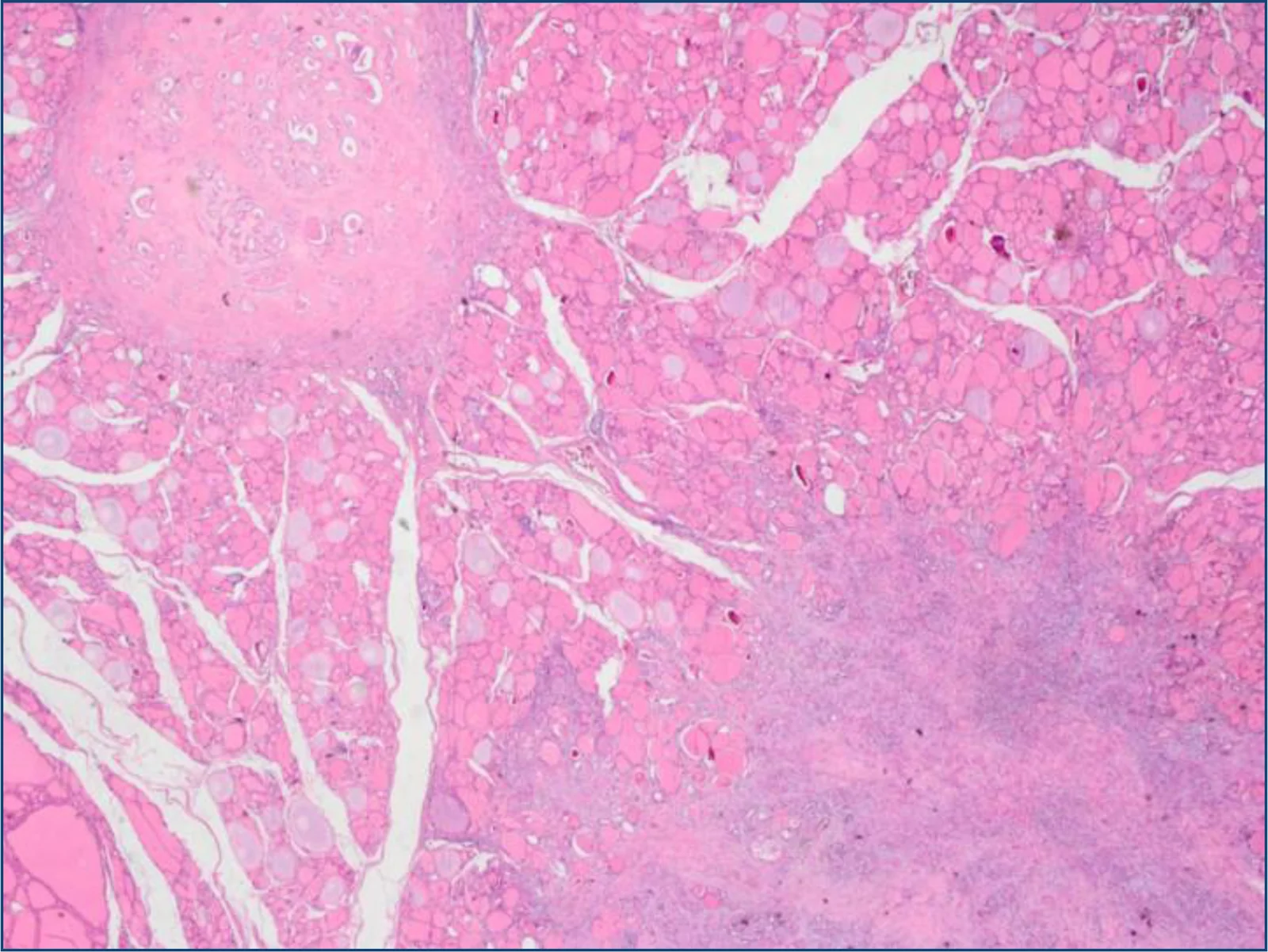
Upper left: subcentimeter classic subtype of papillary thyroid carcinoma. Lower right: focus of subacute granulomatous thyroiditis (hematoxylin & eosin, ×20).

Comparison of clinicopathological parameters between cases with and without malignancy revealed no statistically significant differences in sex, age, thyroid weight, fibrosis score, SGT inflammation score, neutrophilic infiltration, perineural inflammation, intragranulomatous necrosis, lymph node status, chronic lymphocytic thyroiditis, oncocytic change, or vasculitic-like changes (all p>0.05; Table 1).

**Table 1:** Comparison of clinicopathological features according to malignancy status.

Variable		Malignancy present (n=13)	No malignancy (n=31)	p-value
Sex				
	Female	11 (84.6%)	25 (80.6%)	1.000*
	Male	2 (15.4%)	6 (19.4%)	
	Age (years)	48.5±5.7	48.0±10.0	0.813**
	Thyroid weight (g)	29.1±13.2	28.3±15.0	0.878**
Fibrosis score				
	0	1 (7.7%)	3 (9.7%)	0.775*
	1	3 (23.1%)	4 (12.9%)	
	2	3 (23.1%)	5 (16.1%)	
	3	6 (46.2%)	19 (61.3%)	
SGT inflammation score†				
	Score 1 (Mild, <30%)	6 (46.2%)	11 (35.5%)	0.713*
	Score 2 (Moderate, 30–74%)	4 (30.8%)	9 (29.0%)	
	Score 3 (Severe, ≥75%)	3 (23.1%)	11 (35.5%)	
	Neutrophilic infiltration: present	8 (61.5%)	20 (64.5%)	1.000*
	Perineural inflammation: present	8 (61.5%)	20 (64.5%)	1.000*
	Intragranulomatous necrosis: present	1 (7.7%)	1 (3.2%)	0.508*
	Incidental lymph node: present	7 (53.8%)	10 (32.3%)	0.309*
	Chronic lymphocytic thyroiditis: present	4 (30.8%)	3 (9.7%)	0.170*
	Oncocytic change: present	3 (23.1%)	2 (6.5%)	0.144*
	Vasculitic-like changes: present	5 (38.5%)	11 (35.5%)	1.000*

* Fisher's exact test;

** Student's t-test.

† SGT inflammation score: Score 1: <30%; Score 2: 30–74%; Score 3: ≥75% parenchymal involvement. Continuous variables: mean±SD; categorical variables: n (%). SGT: subacute granulomatous thyroiditis.

## DISCUSSION

SGT is traditionally regarded as a self-limited inflammatory disorder with well-defined clinicopathological features^1^. However, the findings of the present study suggest that SGT may have broader diagnostic and pathophysiological implications than previously appreciated. By systematically evaluating 44 thyroidectomy specimens, we identified three key aspects that merit particular attention: (i) the potential role of neural inflammation in pain pathogenesis, (ii) the unexpectedly high rate of coexisting thyroid carcinoma, and (iii) significant limitations of preoperative cytological diagnosis.

The most striking finding was the high frequency of peripheral nerve inflammation, observed in 63.6% of cases. Pain is the most prominent clinical feature of subacute granulomatous thyroiditis. The pain occurring in the thyroid gland is generally attributed to stretching of the thyroid capsule due to inflammation and enlargement of the gland^2–4^. However, this explanation does not fully account for cases with severe pain in the absence of marked enlargement^2^. Our findings provide histopathological support for an alternative or complementary mechanism: direct neural involvement by inflammatory infiltrates. Perineural inflammation has been implicated in pain generation in various inflammatory and neoplastic conditions^5,6^, yet its role in thyroid pathology remains underexplored. Neural involvement has rarely been addressed in previous histopathological studies of SGT, making this observation a potentially underrecognized feature of the disease and warranting further investigation through clinicopathological correlation studies. However, we were unable to perform preoperative pain assessments due to the time elapsed since the surgeries and our inability to reach a significant portion of the patients. Therefore, it was not possible for us to establish a correlation between pain and the severity of perineural inflammation.

Another notable finding is the relatively high prevalence of coexisting thyroid malignancies (29.5%), predominantly subcentimeter papillary thyroid carcinomas. At first glance, this rate appears to exceed those reported in the literature and could raise the question of a potential pathogenetic link between SGT and thyroid carcinoma^7^. However, several lines of evidence argue against a causal relationship. First, the majority of detected malignancies were subcentimeter papillary thyroid carcinomas, many of which were incidental findings. Second, no histopathological transition or spatial association suggesting progression from inflammatory lesions to carcinoma was observed. Third, well-established risk modifiers such as multinodular goiter and chronic lymphocytic thyroiditis were frequently present in the background thyroid tissue. Taken together, we interpret this finding as most likely reflecting incidental detection related to extensive histopathological sampling inherent to total thyroidectomy specimens, rather than a true pathogenetic link. Notably, the observed malignancy rate is comparable to the incidental carcinoma rates routinely encountered in our institution^8,9^. In this regard, SGT may paradoxically increase the detection of incidental carcinomas by prompting more meticulous sampling due to its irregular and suspicious gross appearance.

From a surgical pathology perspective, these findings also have important practical implications. Because SGT is rarely encountered in thyroidectomy specimens, its histopathological spectrum may be unfamiliar to pathologists who infrequently evaluate such cases. Moreover, the irregular gross appearance and extensive inflammatory changes may mimic thyroid malignancy or obscure coexisting neoplastic lesions. Therefore, careful gross examination, adequate histopathological sampling, and thorough microscopic evaluation are essential to establish the correct diagnosis and to avoid overlooking incidental thyroid lesions. The relatively high frequency of incidental carcinoma observed in our series is most likely attributable to extensive histopathological sampling rather than a true biological association. Accordingly, these findings should not be interpreted as evidence that SGT represents a preneoplastic condition.

The limited diagnostic utility of FNAC is a further important finding. Despite being performed in 68.2% of cases, FNAC correctly suggested SGT in only one instance. This low diagnostic yield is likely because FNAC is typically directed toward dominant or suspicious nodules rather than the inflamed parenchyma, thereby missing characteristic granulomatous lesions. Among patients undergoing FNAC, seven were reported as suspicious for malignancy (Bethesda V) and five as follicular neoplasm (Bethesda IV), directly precipitating surgery. These data highlight a critical limitation of FNA in this setting and suggest that SGT may be substantially underrecognized preoperatively, particularly in the presence of coexisting nodular disease^10^. Re-evaluation of cytological specimens or repeat FNAC may help avoid unnecessary surgical intervention^11,12^.

Thyroidectomy is rarely indicated for SGT; NSAIDs are first-line therapy, with corticosteroids reserved for severe or refractory symptoms^13^. Nevertheless, a small number of cases requiring surgery because of severe pain attributable to SGT have been reported in the literature. These reports suggest that thyroidectomy may be considered in selected patients with medically refractory disease and persistent symptoms despite appropriate medical treatment^14,15^. Interestingly, one patient in our cohort underwent surgery with a preoperative diagnosis of SGT after experiencing persistent symptoms despite intermittent corticosteroid therapy for approximately two years. This case further supports the notion that surgical intervention may occasionally have a role in the management of treatment-resistant SGT.

Vasculitic-like changes were identified in 36.4% of cases. Previous reports have suggested that vasculitic processes may play a role in the pathogenesis of SGT, particularly in association with systemic vasculitic disorders such as granulomatosis with polyangiitis, Takayasu arteritis, and Henoch-Schönlein purpura^16,17^. None of our patients had documented systemic vasculitis; nonetheless, these findings suggest that vascular immune-mediated mechanisms may contribute to the inflammatory process in a subset of patients.

A major strength of the present study is the evaluation of a relatively large series of surgically treated SGT cases, a condition that is only rarely managed by thyroidectomy. Comprehensive histopathological re-review of all available specimens enabled systematic assessment of both classical and less commonly described features, including perineural inflammation and vasculitic-like changes, as well as coexisting benign and malignant thyroid lesions.

Nevertheless, several limitations should be acknowledged. The retrospective design and restriction to surgically treated cases introducing inherent selection bias—the observed frequencies, particularly regarding coexisting malignancy, may not reflect true prevalence in the general SGT population. The absence of a control group limited assessment of the specificity of neural and vasculitic findings. Pain severity could not be retrospectively quantified, precluding direct correlation with perineural inflammation. The relatively limited sample size may have reduced statistical power for subgroup analyses.

In conclusion, the frequent presence of perineural inflammation represents a previously underrecognized histopathological finding that may contribute to the pathogenesis of thyroid pain in SGT. Our findings further suggest that the apparent association with thyroid carcinoma is most likely attributable to incidental detection resulting from extensive histopathological sampling rather than a true biological relationship. From a diagnostic perspective, SGT may present significant challenges because its gross appearance can mimic malignancy and obscure coexisting lesions. Therefore, careful gross examination, adequate histopathological sampling, and thorough microscopic evaluation of thyroidectomy specimens are essential to ensure accurate diagnosis and to identify incidental coexisting thyroid neoplasms.

## Data Availability

The datasets generated and/or analyzed during the current study are available from the corresponding author upon reasonable request.
